# The inhibition of Nrf2 accelerates renal lipid deposition through suppressing the ACSL1 expression in obesity-related nephropathy

**DOI:** 10.1080/0886022X.2019.1655450

**Published:** 2019-09-05

**Authors:** Yinyin Chen, Liyu He, Yiya Yang, Ying Chen, Yanran Song, Xi Lu, Yumei Liang

**Affiliations:** a Department of Nephrology, Laboratory of Kidney Disease, Hunan Provincial People's Hospital, Hunan Normal University, Changsha, Hunan, P.R. China;; b Key Lab of Kidney Disease and Blood Purification in Hunan, Department of Nephrology, The Second Xiangya Hospital Central South University, Changsha, Hunan, People's Republic of China

**Keywords:** Nrf2, Oxidative stress, lipid deposition, ACSL1, obesity-related nephropathy

## Abstract

**Background:** Obesity has become a worldwide epidemic, and the incidence of obesity is increasing year by year. Obesity-related nephropathy (ORN) is a common kidney complication of obesity. Long-chain acyl-CoA synthetases-1, (ACSL1), is a key enzyme in the oxidative metabolism of fatty acids in mitochondria and ACSL1 may play a direct role in renal lipid deposition and promote the progress of ORN. In this study, we focus on the renoprotective role of ACSL1 in ORN.

**Methods:** Electron microscopy, immunohistochemical (IHC) staining, Western blot, and real-time PCR were used to detect the expression of ACSL1and Nrf2 in ORN patients, ob/ob mice and palmitic acid (PA)-treated HK-2 cells. Oil red staining and Elisa Kit were used to detect the intracellular FFA and TG contents in ob/ob mice and PA-treated HK-2 cells. Dihydroethidium (DHE) staining and the MDA/SOD measurement were used to detect the ROS production. In order to demonstrate the role of ACSL1 and the interaction between ACSL1 and Nrf2 in ORN, related siRNA and plasmid were transfected into HK-2 cells.

**Results:** More ROS production and renal lipid deposition have been found in ORN patients, ob/ob mice and PA-treated HK-2 cells. Compared with control, all the expression of ACSL1and Nrf2 were down-regulated in ORN patients, ob/ob mice and PA-treated HK-2 cells. The Nrf2 could regulate the expression of ACSL1 and the ACSL1 played the direct role in renal lipid deposition.

**Conclusions:** The Nrf2 is inhibited in ORN, resulting more ROS production and oxidative stress. Increased oxidative stress will suppress the expression of ACSL1, which could increase the intracellular FFA and TG contents, ultimately leading to renal lipid deposition in renal tubulars and accelerating the development of ORN.

## Introduction

Obesity has become a worldwide epidemic, and the incidence of obesity is increasing year by year. Obesity will be one of the most serious public health crises of the 21st century [1]. Obesity-related nephropathy (ORN) usually manifests as lipid deposition in the glomerulus and tubules in patients with body mass index of ≥30 kg/m^2^, is the most accurately described type of renal disease in obese individuals [2,3]. Clinical characteristics of individuals with ORN typically manifest with nephrotic or subnephrotic proteinuria, accompanied by renal insufficiency [4]. The mechanisms involved in ORN are complicated, including chronic inflammation, oxidative stress, insulin resistance, apoptosis etc. [5,6]. Although the disease progressed slowly, ORN has been an increasing reason for the development of end-stage renal disease (ESRD). Over one-third of the patients with ORN have been reported to develop progressive renal failure and ESRD [7]. In recent years, a large number of studies have been focused on ORN, however, the underlying pathophysiological mechanism of ORN is still poorly understood.

Schneider et al. [8] has been demonstrated that fatty acid transport and uptake disorder in kidney is highly relevant for the renal lipid deposition. Renal lipid deposition is a crucial pathological change in ORN and inhibiting renal lipid deposition could slow the progression of ORN [9]. Storage of fatty acid as triglyceride (TG) requires the activation of fatty acids to long-chain acyl-CoAs (LC-CoA) by the enzyme acyl-CoA synthetase (ACSL). There are five known isoforms of ACSL (ACSL1, −3, −4, −5, −6), which vary in their tissue specificity and affinity for fatty acid substrates [10]. Long chain acyl-CoA synthetases-1, (ACSL1), is a key enzyme in the oxidative metabolism of fatty acids in mitochondria. ACSL1 not only could activate fatty acids for intracellular metabolism but are also involved in the regulation of uptake [11]. ACSL1 has been reported in fatty liver, skeletal muscle lipid degeneration, and ACSL is involved in lipid metabolism in different cells, either increasing lipid deposition or promoting lipid catabolism [12,13]. In kidney, inhibition of ASCL1 would result lipotoxic, finally expediting proximal tubule apoptosis [3,9]. Based on these data, we believe ACSL1 may be a key role in the progression of ORN. Interestingly, recent studies have emphasized the association of oxidative stress (ROS) with the pathogenesis of metabolic disorders in obesity [14]. ROS production was thought to be key importance in obesity-related kidney disease [15]. In addition, Trindade de Paula et al. confirmed that ROS levels were opposite to ACSL1 levels [16]. So, ROS production may be involved in the ORN through inhibiting the ACSL1 expression.

As described above, ROS production was thought to be key importance in obesity-related kidney disease. NF-E2-related factor 2 (Nrf2) was generally thought to be a crucial cellular defense against oxidative stress [12,13]. Nrf2 plays a central part in basal activity and coordinates multiple genes [17]. Nrf2 could regulate the expression of antioxidant proteins, which finally results in protecting against oxidative damage [18]. Under normal conditions, Nrf2 is kept in the cytoplasm by Kelch like-ECH-associated protein 1 (Keap-1) [19,20]. Oxidative stress disrupts critical cysteine residues in Keap-1, translocating Nrf2 into the nucleus, where Nrf2 binds to the antioxidant response element (ARE) in the upstream promoter region of many antioxidative genes and initiates their transcription [21]. Activation of Nrf2 could induce many cytoprotective proteins such as (HO-1), an enzyme that catalyzes the degradation of heme into the antioxidant biliverdin, the anti-inflammatory agent carbon monoxide, and ferrous iron [21].These findings demonstrated that Nrf2 have been involved in cellular defense against oxidative stress.

In view of all the findings, this study was initiated to assess whether Nrf2 was inhibited in ORN, which lead to increased oxidative stress. Increased oxidative stress then attenuated the expression of ACSL1, finally resulting more renal lipid deposition and promoting the development of ORN. In conclusion, it is plausible that the Nrf2/ACSL1 signaling pathway plays a critical role in ORN development.

## Materials and methods

### Kidney morphological analysis

Human kidney biopsy tissues were obtained from ORN patients (*n* = 14) or nephritis patients (*n* = 12, control). These patients were confirmed to have pathological and clinical findings consistent with ORN. Renal biopsy tissue sections were subjected to immunohistochemical (IHC) staining for Nrf2 and ACSL. The renal lipid deposition was analysis by electron microscopy. The study protocol was approved by the Ethics Committee of The Second Xiangya Hospital, Central South University and all human samples were collected with the patient’s consent.

### Animals

Twelve-week-old male C57BL/6 J ob/ob and C57BL/6 J ob/m mice were used for the animal experiments. These mice were purchased from the JunKe Experimental Animal Company (Nanjing, China). They were organized into the following two groups for the animal experiments: ob/m group (control, *n* = 6) and a ob/ob group (*n* = 6).The mice were euthanized at 14 weeks of age. The Animal Care and Use Committee of Second Xiangya Hospital of Central South University approved all animal procedures.

### Cell culture

Human proximal tubular epithelial cells (HK-2) were cultured in Dulbecco’s modified Eagle’s medium (Sigma-Aldrich) supplemented with 10% fetal bovine serum, 0.5% penicillin and streptomycin in 5% CO_2_ incubator at 37 °C. For transfection experiment, transfection of siRNA (100 nM) or plasmid (2500 ng), and then the HK-2 cells were treated with or without palmitic acid (PA) (0.04 mmol/l) for 24 h [22,23].

### Extraction of total RNA and quantitative real-time PCR

Total RNA was isolated from the kidneys of individual mice or patients using TRIzol (TaKaRa, Dalian, China). cDNA was synthesized using the M-MLV Reverse Transcriptase cDNA Synthesis Kit (TaKaRa) according to the manufacturer’s instructions. Real-time PCR was performed with an ABI Prism 7300 Sequence Detection system (Applied Biosystems) using the SYBR^®^ Premix Ex Taq TM II (TaKaRa). The gene expression in each sample was analyzed in duplicate and normalized against the internal control gene GAPDH. Relative quantification of the target gene expression in patients compared with normal samples was performed with the ΔΔCt method

### Western blotting

Lysates of kidney tissue or HK-2 cells were prepared. All the antibodies in western blot were purchased from Cell Signaling Technology Inc. (Beverly, MA) and the dilute concentration is based on antibody instruction. After centrifugation, the protein level was determined in supernatants using a Micro BCA protein assay kit with BSA as a standard (Pierce, Thermo). In brief, 30 μg protein was used for electrophoresis on SDS-PAGE and transferred to a nitrocellulose fibrous membrane. The blots were blocked in 5% nonfat dry milk for 1 h, followed by overnight incubation at 4 °C with the following primary antibodies, respectively, Nrf2: (rabbit anti-human, 1:1000 Abcam, USA), ASCL1 (rabbit anti-Human, 1:1000; Abcam). Rabbit anti-human β-actin-specific antibody (1:1 000; Abcam, Cambridge, UK) was used for loading controls on stripped membranes. After being washed with TBS, blots were incubated with an HRP-conjugated secondary antibody (goat anti-rabbit, 1:1 000 Vectastain Elite; Vector Laboratories, Peterborough, UK) at room temperature for 1 h, and then enhanced chemiluminescence (Thermo, Rockford, IL) was used to visualize the bands.

### Measurement of ROS generation

Dihydroethidium (DHE) was used to assess the production of intracellular superoxide anions(O2-). Malondialdehyde (MDA) formation was utilized to quantify a naturally occurring product of lipid peroxidation and measured as thiobarbituric acid-reactive material. Kidney tissue MDA levels were quantified with a commercially available assay (Northwest Life Science Specialties, Vancouver, WA). The results are expressed as nanomoles MDA per gram tissue. Prior to biochemical analysis, kidneys from each group were also homogenized and used for the analysis of enzymatic as well as nonenzymatic antioxidants. Superoxide dismutase (SOD) activity was measured by an ELISA kit (R&D Systems), the procedure was according to the manufacturer’s instructions.

### Lipid measurement

Quantitative measurement of FFA and TG levels in cells and kidneys were performed using an enzyme-linked immunosorbent assay (Cusabio Biotech Co. Ltd, Wuhan, China).

### Oil red O staining

For lipid analysis, frozen sections of kidneys were fixed and stained with Oil Red O for 15–30 min, and samples, after being washed, were then stained with hematoxylin for another 2 min. All images were captured using a fluorescence microscope (Nikon, Tokyo, Japan). All IHC were repeated at least three times and representative images were presented.

### Immunohistochemistry

Immunohistochemical studies were performed on sections of formalin-fixed and paraffin-embedded kidney tissues. Endogenous peroxidases were inactivated using 3% H_2_O_2_, followed by blocking with goat serum. Sections were incubated overnight (4 °C) with anti-Nrf_2_ (1:200; Abcam) and anti-ACSL1 (1:200; Abcam). Then, the sections were washed and incubated for 45 min with secondary antibody. Histochemical reactions were performed using a DAB kit, and sections were counterstained with hematoxylin. The Images were acquired from a fluorescence microscope (Nikon, Tokyo, Japan). All IHC were repeated at least three times and representative images were presented.

### Statistical analyses

Data are expressed as means ± standard error (SE). Comparisons between two groups were evaluated by ANOVA a post hoc test. Values of *p* < 0.05 were regarded as significant. The above analyses were conducted with GraphPad Prism 5.0 and SPSS 17.0 statistical software.

## Results

### Increased renal lipid deposition in renal biopsy in patients with ORN

**Figure 1.**
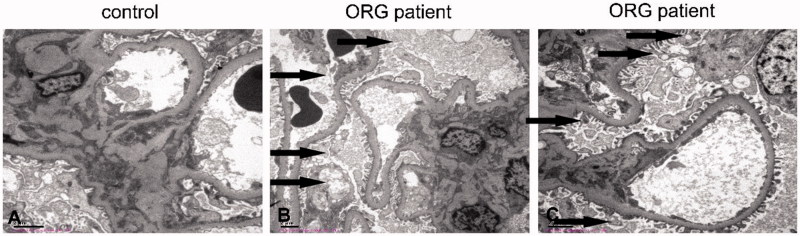
Increased renal lipid deposition in renal biopsy in patients with ORN. Electron microscopy analysis showed lipid accumulation in podocytes (B) and mesangial cells (C) in ORN patients, while not in the nephritis ones (A).

The clinical characteristics of the ORN patients and nephritis patients, who served as controls in this study, are shown in Table 1. ORN patients exhibited significantly increased in total cholesterol and TG levels, as well as increased 24 h urine protein excretion, compared with control subjects. There were no significant differences between ORN patients and control subjects with respect to age, sex. Furthermore, electron microscopy analysis showed lipid accumulation in podocytes (Figure 1(B)) and mesangial cells (Figure 1(C)) in ORN patients. These data indicated that renal lipid deposition may play a crucial role in the progression of ORN.

**Table 1.** Baseline characteristics of ORG and control participants.

Variables	ORG	Control
Number	14	12
Sex (m/f)	8/6	7/5
Age (years)	34.45 ± 5.44	33.66 ± 4.17
TC (mmol/L)	6.26 ± 0.54*	3.82 ± 0.61
TG (mmol/L)	2.89 ± 0.21*	1.07 ± 0.14
Serum creatinine (μmol/L)	135 ± 6.7*	57 ± 6.4
Blood urea nitrogen (mmol/L)	37.15 ± 4.12*	5.7 ± 0.54
24-h proteinuria (mg)	1519 ± 100.72*	101 ± 9.81*
Systolic blood pressure (mmHg)	151.64 ± 9.19*	115.61 ± 5.12
Diastolic blood pressure (mmHg)	96.14 ± 8.17*	76.56 ± 8.13

m: male; f: female; TC: total cholesterol; TG: triglyceride *p < 0.05 versus control; values are means ± SEM.

### Renal ACSL1and Nrf2 expressions were down-regulated in ORN patients

**Figure 2.**
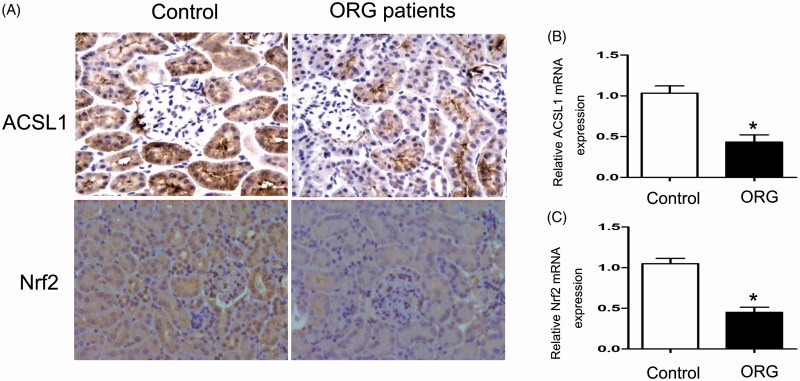
Renal ACSL1 and Nrf2 expressions were down-regulated in ORN patients. (A) Immunohistochemistry for ACSL1 and Nrf2, 200×. (B, C) Real-time PCR for ACSL1 and Nrf2. Total RNA was isolated from the renal biopsy of individual using TRIzol; Values are the mean ± SEM, *p < 0.05, compared with control.

Immunohistochemical staining and real-time PCR were used to detect the expressions of ACSL1and Nrf2 in ORN patients. IHC staining demonstrated significantly decreased ACSL1and Nrf2 expressions in ORN renal biopsy compared with control (Figure 2(A)). These findings were also confirmed by RT-PCR (Figure 2(B,C)). These data indicated the crucial role of Nrf2/ACSL1 pathway in ORN.

### Oxidative stress and renal total lipid were increased in ob/ob mice

**Figure 3.**
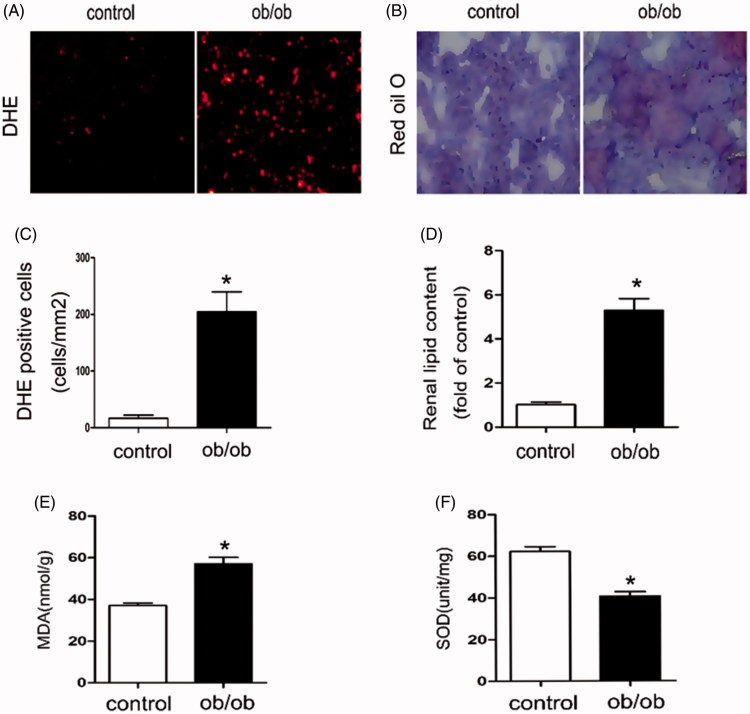
The oxidative stress and renal total lipid were increased in ob/ob mice. Kidney samples were collected from ob/ob mice or C57BLK mice (control). Thebaseline characteristics of these mice were shown in Table 2. (A) DHE staining of kidney tissue, 200×; (B) Oil Red O-stained kidney sections, 400×. (C) Statistics analysis of DHE positive cells. Values are the mean ± SE, *p < 0.05, compared with control; (D) Quantitative measurement of FFA and TG contents in the kidneys. Values are the mean ± SE, *p < 0.05, compared with control; (E) Determination of MDA levels in Serum by ELISA Kit, Values are the mean ± SE, *p < 0.05, compared with control; (F) Determination of SOD levels in Serum by ELISA Kit, Values are the mean ± SE, *p < 0.05, compared with control.

The baseline characteristics of ob/ob mice and control mice were shown in Table 2. Compared with control mice, both of the serum TG and serum FFA were increased in ob/ob mice, as well as increased 24 h urine protein excretion. Reactive oxygen species (ROS) generation was increased in the renal of ob/ob mice compared with C57BLK (control) mice, as shown by staining with DHE, an indicator of oxidation (Figure 3(A–C)). Likewise, in ob/ob mice serum, malondialdehyde (MDA) levels were up-regulated (Figure 3(E)) and the superoxide dismutase (SOD) levels were down-regulated (Figure 3(F)) compared with control mice. These data verified that in ob/ob mice kidney, the oxidative stress was activated. Besides, Oil Red O staining and TG/FFA quantitation revealed much more lipid accumulation in the glomeruli and tubules of the kidneys of ob/ob mice than those of control mice (Figure 3(B–D)), suggesting that more lipids may relocate to the kidneys of obese mice.

**Table 2.** Baseline characteristics of ob/ob mice and control mice.

Variables	ob/ob	Control
Serum TG (mmol/l)	1.58 ± 0.17*	0.86 ± 0.12
Serum FFA (mmol/l)	2.01 ± 0.23*	0.81 ± 0.0
Proteinuria (mg/day)	467.4 ± 20.1*	18.2 ± 2.7
Weight (g)	43.8 ± 4.1*	23.2 ± 2.9
Serum creatinine (mg/dL)	0.75 ± 0.12*	0.48 ± 0.16
Blood urea nitrogen (mg/dL)	46.15 ± 7.67	39.47 ± 5.16

### Renal ACSL1and Nrf2 expressions were down-regulated in ob/ob mice

**Figure 4.**
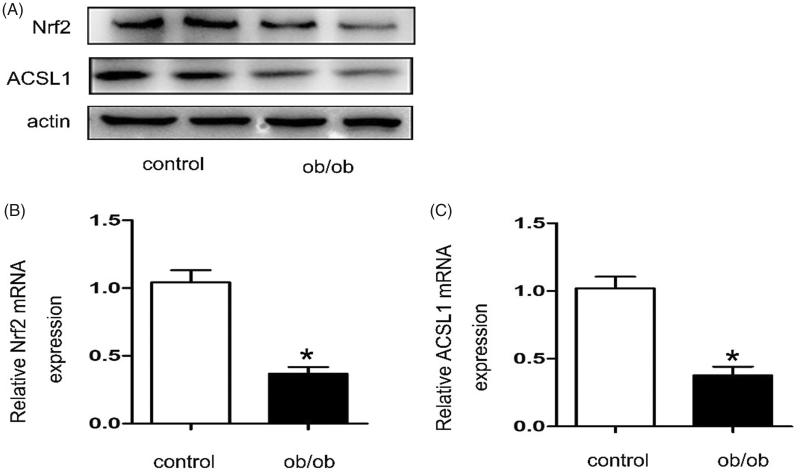
Renal ACSL1 and Nrf2 expressions were down-regulated in ob/ob mice. Kidney samples were collected from ob/ob mice or C57BLK mice (control). The baseline characteristics of these mice were shown in Table 2. (A) Western blot for ACSL1 and Nrf2. (B, C) Real-time PCR for ACSL1 and Nrf2. Total RNA was isolated from the kidneys of individual mice using TRIzol; Values are the mean ± SEM, *p < 0.05, compared with control.

Western blot and real-time PCR were used to detect the expression of ACSL1 and Nrf2 in ob/ob mice. As shown in Figure 4, compared with control mice, the expressions of ACSL1 and Nrf2 were down-regulated in ob/ob mice (Figure 3(A)). These findings were also confirmed by RT-PCR for kidney tissues (Figure 4(B,C)). Importantly, the animal experiment results were consistent with the clinical result.

### The oxidative stress and lipid content increased in PA-treated HK-2 cells

**Figure 5.**
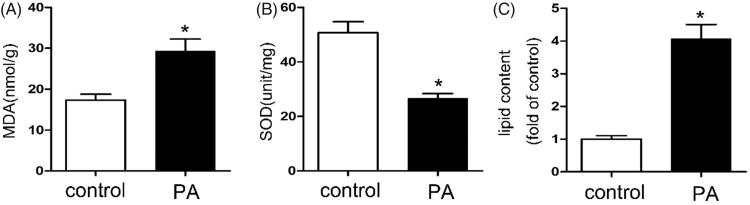
The oxidative stress and lipid content increased in PA treated HK-2 cells. Human proximal tubular epithelial cells (HK-2) were cultured in Dulbecco’s modified Eagle’s medium (Sigma-Aldrich) supplemented with 10% fetal bovine serum, 0.5% penicillin and streptomycin in 5% CO2 incubator at 37° C. And then the HK-2 cells were treated with or without palmitic acid (PA) (0.04 mmol/l) for 24 h. (A) Determination of MDA levels by ELISA Kit, Values are the mean ± SE, *p < 0.05, compared with control; (B) Determination of SOD levels by ELISA Kit, Values are the mean ± SE, *p < 0.05, compared with control; (C) Quantitative analysis of intracellular FFA and TG contents in HK2 cells. Data are expressed as mean ± SEM from four independent experiments. *p < 0.05 versus the control group.

Reactive oxygen species (ROS) generation was increased in PA treated HK-2 cells compared with control. As shown in Figure 5, the MDA levels were up-regulated (Figure 5(A)) and the SOD levels was down-regulated (Figure 5(B)) compared with control group. Besides, the quantitative analysis of cellular TG and FFA revealed increased lipid accumulation in HK2 cells after PA treatment (Figure 5(C)). These results suggest that the oxidative stress and lipid content increased in PA treated HK-2 cells

### The expressions of ACSL1 and Nrf2 were down-regulated in PA-treated HK-2 cells

**Figure 6.**
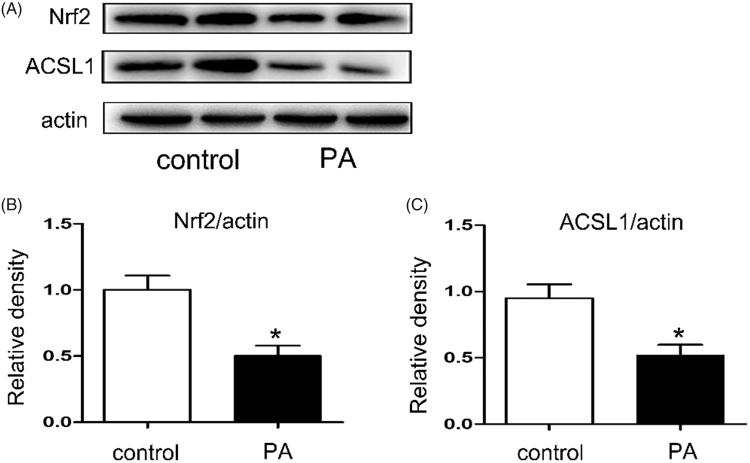
The expressions of ACSL1 and Nrf2 were down-regulated in PA treated HK-2 cells. Human proximal tubular epithelial cells (HK-2) were cultured in Dulbecco’s modified Eagle’s medium (Sigma-Aldrich) supplemented with 10% fetal bovine serum, 0.5% penicillin, and streptomycin in 5% CO2 incubator at 37° C. And then the HK-2 cells were treated with or without palmitic acid (PA) (0.04 mmol/l) for 24 h. (A) Western blot for ACSL1 and Nrf2; (B, C) Densitometry analysis is presented as relative ratios of Nrf2/actin and ASCL1/actin. The data are means ± SE (*n* = 4); *p < 0.05 compared with control.

Western blot was used to detect the expressions of ACSL1 and Nrf2 in PA-treated HK-2 cells. Human proximal tubular epithelial cells (HK-2) were cultured in Dulbecco’s modified Eagle’s medium (Sigma-Aldrich) supplemented with 10% fetal bovine serum, 0.5% penicillin, and streptomycin in 5% CO_2_ incubator at 37 °C. And then the HK-2 cells were treated with or without PA (0.04 mmol/l) for 24 h. As shown in Figure 6, compared with control, the expressions of ACSL1 and Nrf2 were all down-regulated in PA-treated HK-2 cells (Figure 6(A)).

### The ACSL1 expression was regulated by Nrf2

**Figure 7.**
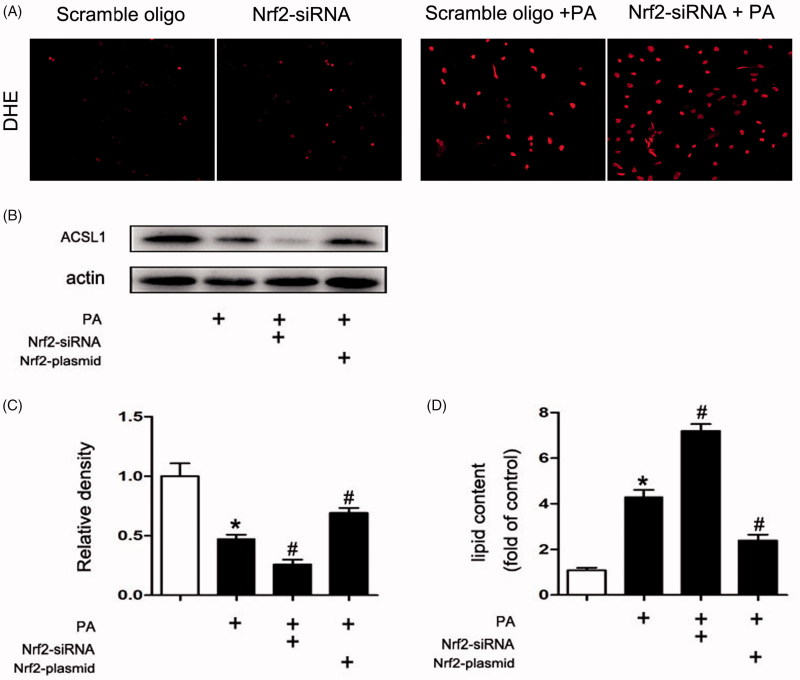
The ACSL1 expression was regulated by Nrf2. Human proximal tubular epithelial cells (HK-2) were cultured in Dulbecco’s modified Eagle’s medium (Sigma-Aldrich) supplemented with 10% fetal bovine serum, 0.5% penicillin and streptomycin in 5% CO2 incubator at 37 °C. For transfection experiment, transfection of siRNA (100 nM) or plasmid (2500 ng), and then the HK-2 cells were treated with or without palmitic acid (PA) (0.04 mmol/l) for 24 h. (A) DHE staining of HK2 cells 200×. (B) Western Blot for ACSL1. (C) Densitometry analysis is presented as relative ratios of ASCL1/actin. The data are means ± SE (*n* = 4); *p < 0.05 compared with control; #*p* < 0.05 compared with PA group. (D) Quantitative analysis of intracellular FFA and TG contents in HK2 cells. The data are means ± SE (*n* = 4). *p < 0.05 versus the control group. #*p* < 0.05 compared with PA group.

As described before, increased ROS production will inhibit ACSL1 expression. Our results indicate that inhibition Nrf2 by Nrf2-siRNA will increase ROS production in PA-treated HK2 cells (Figure 7(A)). In order to further verify the role of Nrf2 in the expression of ACSL1.The HK-2 cells were transfected with Nrf2-siRNA or Nrf2-plasmid to inhibit or overexpress Nrf2. Compared with control, the expression of ACSL1 in HK-2 cells was down-regulated after PA treatment. Besides, compared with treatment with PA alone, more pronounced inhibition of ACSL1 synthesis was obtained when the cells were transfected with Nrf2-siRNA. However, the inhibition will be milder when the cells were transfected with Nrf2 plasmid (Figure 7(B,C)). The opposite result could be obtained to the quantitative analysis of cellular TG and FFA (Figure 7(D)).

### ACSL1 was the direct role in renal lipid deposition in ORN

**Figure 8.**
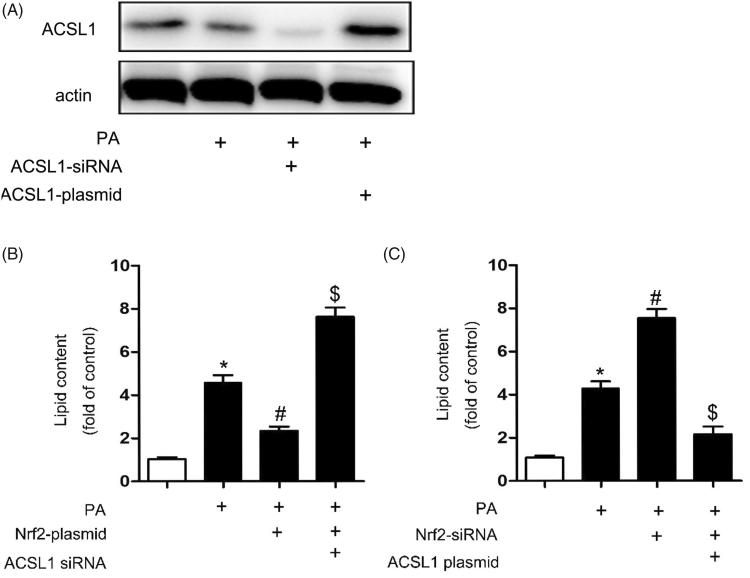
ACSL1 was the direct role in renal lipid deposition in ORN. Human proximal tubular epithelial cells (HK-2) were cultured in Dulbecco’s modified Eagle’s medium (Sigma-Aldrich) supplemented with 10% fetal bovine serum, 0.5% penicillin and streptomycin in 5% CO2 incubator at 37° C. For transfection experiment, transfection of siRNA (100 nM) or plasmid (2500 ng), and then the HK-2 cells were treated with or without palmitic acid (PA) (0.04 mmol/l) for 24 h. (A) Western blot for ACSL1. (B) Quantitative analysis of intracellular FFA and TG contents in HK2 cells. Data are expressed as mean ± SE from four independent experiments. *p < 0.05 versus the control group; #*p* < 0.05 versus the PA group. $*p* < 0.05 versus the PA + Nrf2-plasmid group. (C) Quantitative analysis of intracellular FFA and TG contents in HK2 cells. Data are expressed as mean ± SE from four independent experiments. *p < 0.05 versus the control group; #*p* < 0.05 versus the PA group. $*p* < 0.05 versus the PA + Nrf2-siRNA group.

In order to verify the role of Nrf2 and ACSL1 in renal lipid deposition, the HK-2 cells were transfected with Nrf2-siRNA, ACSL1-plasmid, Nrf2-plasmid or ACSL1-siRNA. We first verified that ACSL1-siRNA could inhibit ACSL1 expression and ACSL1-plasmid could promote ACSL1 expression in PA-treated HK2 cells (Figure 8(A)). Then, our results indicated that, compared with control, the lipid contents were up-regulated after PA treatment. Besides, compared with treatment with PA alone, there is only a slight up-regulation when the cells were transfected with Nrf2-plasmid before. But dramatic increase will be obtained when the cells were transfected with Nrf2-plasmid and ACSL1-siRNA simultaneously before (Figure 8(B)). In contrast, dramatic increase will be obtained when the cells were transfected with Nrf2-siRNA compared with treatment with PA alone. However, only slight up-regulation will be found if the cells were transfected with Nrf2-siRNA and ASCL1 plasmid before (Figure 8(C)). These results indicated that the ACSL1 was the direct role in renal lipid deposition in ORN.

## Discussion

The purpose of our current study was to clarify the role of ACSL1 in ORN. Here we show that: (1) oxidative stress increased in ORN patients, ob/ob mice and PA treated HK-2 cells (Figures 3 and 5); (2) both the expression of ACSL1 and Nrf2 are down-regulated in ORN patients, ob/ob mice and PA-treated HK-2 cells (Figures 2, 4, and 6); (3) The expression of ACSL1 is mainly regulated by Nrf2 (Figure 7); and (4) ACSL1 is the key role for renal lipid deposition in ORN (Figure 8). Taking all the findings together, we believe oxidative stress increases renal lipid deposition mainly through suppressing the expression of ACSL1 in ORN, and the increased oxidative stress in ORN mainly caused by the inhibition of Nrf2. So, we have provided a Nrf2/ACSL1 pathway in ORN, which may provide the potential therapeutic target for ORN patients.

Fatty acid homeostasis is regulated by a complex system. Lipid metabolism imbalance will lead to lipid ectopic deposition, resulting in lipotoxicity [22]. Cellular lipotoxicity, which involves the cellular accumulation of nonesterified FFA and TG, is thought to contribute to ORN [24]. The “lipid nephrotoxicity hypothesis” was first proposed by Moorhead et al., which stimulated large studies of the relationship between lipids and renal disease [22,25]. Accumulated evidences suggested a direct role of lipids in the initiation and progression of ORN [22,26]. Besides, there is sufficient evidence that ectopic lipid is associated with structural and functional changes of renal mesangial and epithelial cells to propose the development of obesity-related kidney disease [22,27]. Based on these findings, we believe renal lipid deposition is a crucial pathological change in ORN and inhibiting renal lipid deposition could slow the progression of ORN. In our study, the intracellular FFA and TG contents in both ob/ob mice and PA treated HK-2 cells were up-regulated, indicating renal lipid deposition have been involved in the pathogenesis of ORN (Figures 3 and 5).

ACSL1, is a key enzyme in the oxidative metabolism of fatty acids in mitochondria. ACSL1 not only could activate fatty acids for intracellular metabolism but also are involved in the regulation of uptake [10,11]. Lack of ACSL1 will induce fatty acids utilization disorders, which finally could lead to ectopic deposition of lipids [28,29]. In our study, compared with control, the expression of ACSL1 was down-regulated in ORN patients, ob/ob mice and PA treated HK-2 cells (Figures 2, 4 and 6), indicating the crucial role of ACSL1 in the pathogenesis of ORN. Besides, overexpression of ACSL1 will reduce intracellular FFA and TG contents in PA-treated HK-2 cells (Figure 8), which means we could reduce the renal lipid deposition in ORN through increasing the expression of ACSL1, ultimately delaying the progress of ORN.

A large number of evidences suggests that activated Nrf2 exert protective effects by ameliorating the production of ROS and increasing the antioxidant capacity [30]. In CKD, oxidative stress is partly due to a diminished antioxidant capacity which is largely caused by impaired activation of Nrf2 [31]. Interestingly, as described above, ROS levels were opposite to ACSL1 levels [16]. Reducing ROS production will increase the expression of ACSL1 [16,32]. Based on these data, we believe the expression of ACSL1 is regulated by Nrf2. In our study, the expression of Nrf2 was down-regulated in ORN patients, ob/ob mice and PA treated HK-2 cells (Figures 2, 4, and 6), indicating Nrf2 has been involved in the pathogenesis of ORN. Compared with PA treatment alone, more pronounced inhibition of ACSL1 synthesis was obtained when the HK-2 cells were transfected with Nrf2-siRNA. However, the ACSL1 inhibition will be milder when the cells were transfected with Nrf2 plasmid (Figure 7). These findings revealed that ACSL1 expression was regulated by Nrf2. Besides, we also found the intracellular FFA and TG contents will largely increase when we transfected the HK-2 cells with ACSL1 siRNA, whether it was transfected with Nrf2-siRNA or Nrf2-plasmid simultaneously (Figure 8(B)). In contrast, the intracellular FFA and TG contents will significantly decrease when we transfected the HK-2 cells with ACSL1 plasmid, whether it was transfected with Nrf2-siRNA or Nrf2-plasmid simultaneously (Figure 8(C)). This interesting result demonstrated that ACSL1 play the direct role in renal lipid deposition in ORN.

In summary, the Nrf2 is inhibited in ORN, resulting more ROS production and oxidative stress. Increased oxidative stress will suppress the expression of ACSL1, which could increase the intracellular FFA and TG contents, ultimately leading to renal lipid deposition and accelerating the development of ORN. Delineation of the Nrf2/ACSL1 pathway may provide novel therapeutic targets for ORN patients.
